# Dynamic Effect of Operational Regulation on the Mesophilic BioMethanation of Grape Marc

**DOI:** 10.3390/molecules26216692

**Published:** 2021-11-05

**Authors:** Josue Kassongo, Esmaeil Shahsavari, Andrew S. Ball

**Affiliations:** School of Science, RMIT University, Melbourne, VIC 3083, Australia; e.shahsavari@gmail.com (E.S.); andy.ball@rmit.edu.au (A.S.B.)

**Keywords:** anaerobic digestion, grape marc, mesophilic, substrate-to-inoculum ratio, waste management, winery

## Abstract

Wine production annually generates an estimated 11 million metric tonnes of grape marc (GM) worldwide. The diversion of this organic waste away from landfill and towards its use in the generation of renewable energy has been investigated. This study aimed to evaluate the effectiveness of operational parameters relating to the treatment regime and inoculum source in the extraction of methane from GM under unmixed anaerobic conditions at 35 °C. The study entailed the recirculation of a previously acclimated sludge (120 days) as downstream inoculum, an increased loading volume (1.3 kg) and a low substrate-to-inoculum ratio (10:3 SIR). The results showed that an incorporation of accessible operational controls can effectively enhance cumulative methane yield (0.145 m^3^ CH_4_ kg^−1^ VS), corresponding to higher amounts of digestible organics converted. The calculated average volumetric methane productivity equalled 0.8802 L CH_4_ L_Work_^−1^ d^−1^ over 33.6 days whilst moderate pollutant removal (43.50% COD removal efficiency) was achieved. Molecular analyses identified *Firmicutes* and *Bacteroidetes* phyla as core organisms for hydrolytic and fermentative stages in trophic relationships with terminal electron acceptors from the methane-producing *Methanosarcina* genus. Economic projections established that the cost-effective operational enhancements were sustainable for valorisation from grape marc by existing wineries and distilleries.

## 1. Introduction

Grape is an economically significant fruit crop, with over 79 megatons (Mt) produced globally [1,2]. An estimated 75% of the total grape production is crushed for winemaking [3]. At the end of fermentation, wine contains approximately 20% (*w*/*v*) of solid material, known as grape marc (GM) or pomace, which is produced at a rate of 2 t ha^−1^ [4,5,6]. After the primary grape juice has been extracted, the by-product (GM) is traditionally channelled to distilleries for additional valorisation. Resource recovery includes further alcohol, phenol, and tartrate extraction from GM through distillation [3,6]. Additionally, GM has been used as the raw material for the extraction of natural antioxidants, preservatives, and colouring agents for the food industry [7,8,9,10].

Further benefit from GM includes its application in anaerobic digestion (AD) technology to produce bioenergy whilst concurrently achieving waste reduction [11,12,13,14,15,16,17]. High methane yield is the overarching goal in the anaerobic treatment of organics. The main routes to meet this fundamental objective have been through the refining of pre-treatment, physicochemical, biological, and operational parameters. While such factors are regularly presented individually, they are not mutually exclusive. More often than not, a mix of approaches can compound contributory parameters [18,19,20].

Firstly, the hydrolysis of polymers into monomers requires that organic residues be amenable to hydrolytic enzymes. Consequently, an upstream pre-treatment step is often intercalated in the digestion process to enhance the solubilisation of wastes [21,22]. For example, Tian et al. [23] performed sequential ultrasonication (ULS) and ultrasonication-ozonation (ULS-Ozone) pre-treatments on sewage sludge to improve solubilisation; subsequent semi-continuous anaerobic treatment at 35 °C resulted in the generation of 309 and 348 mL gas g^−1^ COD for ULS and ULS-Ozone, respectively. In contrast, the baseline control without pre-treatment was at 256 mL gas g^−1^ COD. Pre-treatment regimes not only increased bioenergy outputs but also halved the solid residence time and presented greater waste remediation, without negatively impacting reactor performance [23]. The pre-treatment strategies can be physical, chemical, biological or an assortment of these [22,24,25,26,27,28]. However, the high costs of acid/alkaline chemicals and their build-up during pre-treatment can be inhibitory to subsequent methane production. The sodium contained in NaOH alkaline pre-treatment solutions was reported to reach 8 g Na L^−1^, inhibiting methanogenesis [27]. Additionally, a positive correlation between the energy input of various physical pre-treatments and the corresponding cumulative methane yield was observed, rendering the bioenergy production uneconomical; mechanical shredding required the least energy input but resulted in only 10% increased biogas production when compared to other energy-consuming methods, such as sonication [27].

Secondly, physicochemical regulatory options include salinity and conductivity adjustments, nutrient addition, and trace metals addition, among others [29,30]. In using nutrient and trace element solutions in the treatment of GM at 35–37 °C, Fabbri et al. [29] reached a biogas production of 405.65 mL gas g^−1^ VS over 34 days. However, without physicochemical adjustments at 40 °C, reactor performance was limited to 250 mL gas g^−1^ VS over 40 days [31]. However, the recurrent drawback in the use of physicochemical enhancers was the environmental impact associated with the use of synthetic materials such as magnetite, iron powder, and granular activated carbon [32,33,34]. Furthermore, the energy balance in most cases has been negative, characterised by incurred costs not compensating for the exergonic outflow.

Thirdly, cases where biological parameterisations were executed in the establishment of reactor performance, shifts in microbial dynamics and community structure were affected by the mixing of different organic types. The ensuing bioaugmentation positively reflected on the bioenergy profile [35,36,37]. For example, *Proteobacteria* were the predominant microorganisms observed in the mono-digestion of sewage sludge. However, when co-digested with urban organic wastes at 55 °C, *Thermonema* increased in abundance whereas the *Proteobacteria* population receded. *Proteobacteria* are hydrolytic, acidogenic, and non-thermophilic bacteria unsuitable for life at high temperatures [38]; in contrast halophilic *Thermonema* have an optimal growth temperature of 60 °C and are endowed with fermentative physiologies for the transformation of monosaccharides into acetate, lactate, and gaseous CO_2_ and H_2_ [35,39]. Despite the shifts in microbial community richness, there was active degradation of polymers [35,36]. Generally, various types of wastes have been used in co-digestion to alter nutritional compositions, thus selecting for the microbial community in the influent, from lignocellulosic materials such as wood and paper, and food wastes, to the organic fraction of municipal solid waste [40,41,42,43,44,45].

Lastly, a combination of operational parameters can be envisaged for treatment. These include organic loading rate (OLR), hydraulic residence time (HRT), working volume, substrate-to-inoculum ratio (SIR), temperature regimes, and slurry homogenisation, among others [46,47,48]. These operational refinements were aimed at reducing the lag period and accelerating the establishment of sustained biogas production. At increasing total solids (10–15% TS), the mixed manure slurry produced 10–30% more biogas than unmixed conditions at 35 °C [46]. Separately, Ma et al. [48] observed that a lower range of SIR (1:2, 1:1, and 2:1) exhibited steady biogas profiles, whereas a higher range SIR (3:1 and 4:1) showed a prolonged lag time of 15–25 days before establishment of biogas production due to the inhibitory accumulation of volatile fatty acids and low pH over a treatment period of 60 days at 37 °C. Wang et al. [49], while digesting grass silage (20% *w*/*w*) and cow manure anaerobically in continuously stirred systems observed a stable microbial community richness. However, when the grass silage mixing ratio was doubled to 40% *w*/*w*, there were large variations in the microbial structure. Additionally, Fitamo et al. [35] reported a decrease in the relative abundance of *Methanothermobacter* and an increase in *Methanosarcina* upon reduction of the HRT from 20 to 15, and 10 days.

Taken together, operational controls integrate digestion parameters (namely pre-treatment, physicochemical, and biological) by impacting on the various key characteristics of the digestate, thus exerting a selective pressure on the bacterial community that ultimately drives biogas output and digester stability. Therefore, this research aimed to achieve bioenergy production by the targeting of operational controls as a multi-level anaerobic digestion performance enhancer. This was achieved by the application of cost-effective operational parameters of the treatment regime (HRT, working volume, and inoculum source) in a single-stage digester using unmixed conditions at 35 °C for the treatment of GM at high total solids, without exogenous chemical enhancers.

## 2. Results and Discussion

### 2.1. Reactor Performance from the Mono-Digestion of Grape Marc

#### 2.1.1. Bioenergy Production

**Figure 1:**
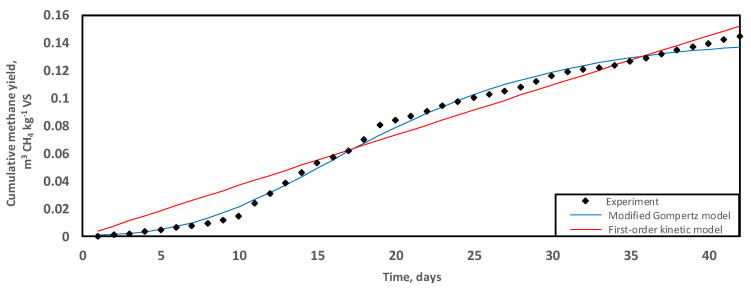
Trends of methane production (m^3^ CH_4_ kg^−1^ VS) during the digestion of grape marc at 35 °C over a period of 42 days. Experimental data fitted with predictive regression models, the modified Gompertz (blue) and first-order kinetic (red).

Anaerobic digestion exhibited a predominantly monophasic, linear methane production curve over a treatment period of 42 days at 35 °C (Figure 1). The maximum cumulative SMY equalled 0.145 ± 0.00 m^3^ CH_4_ kg^−1^ VS. On day 19 of treatment, 55% of the overall methane productivity had been extracted from GM. Similarly, Da Ros et al. [50] did not observe a lag time in the establishment of bioenergy production when active microbial consortia were used to inoculate winery residues. Additionally, there was an improved COD/N nutritional composition and the presence of readily digestible soluble compounds in the residues, which accounted for a shortened reactor start-up [15,50].

The maximum cumulative SMY (0.145 ± 0.00 m^3^ CH_4_ kg^−1^ VS) of the GM mono-digestion for this study was achieved within the limits of a sustainable operation, without requirements for mixing, pH control, and exogenous chemical enhancers. In contrast, Fabbri et al. [29] employed stirred systems supplemented with synthetic nutrients, trace elements, and Na_2_CO_3_ for pH adjustment and produced 0.157 m^3^ CH_4_ kg^−1^ and 0.273 m^3^ CH_4_ kg^−1^ for the *Nero Buono* grape marc and *Greco* grape marc varieties, respectively. However, research translation into a winery-scale energy production would be impeded by the addition of such exogenous enhancers due to restrictive operating costs and environmental considerations [51,52].

#### 2.1.2. Volumetric Methane Productivity Rate (VMPR)

**Figure 2:**
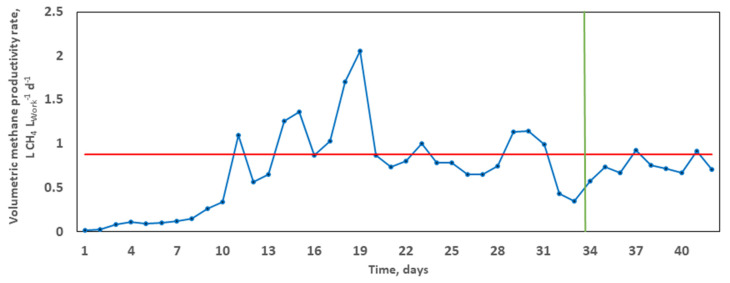
Typical trend of the volumetric methane productivity rate (VMPR) during the grape marc mono-digestion. The blue curve depicts daily variations. The calculated average VMPR (shown in red) at the corresponding time T_80_ (x-intercept, shown in green).

The average specific methane production equalled 38.45 L CH_4_, corresponding to a calculated average VMPR of 0.8802 L CH_4_ L_Work_^−1^ d^−1^. It took 33.6 days (x-intercept) to achieve 80% of the overall methane yield (Figure 2). The volumetric methane productivity rate enables satisfactory comparability across studies based on methane extraction, normalised to the overall substrate volume for treatment. For example, in the co-digestion of rape straw and dairy manure, Ma et al. [48] reached lower VMPR values in the range 0.1–0.5 L CH_4_ L_Work_^−1^ d^−1^, achieving a maximum methane yield of 209.1 mL CH_4_ g^−1^ VS and an SIR of 2:3 over 60 days. It can thus be concluded that, at a higher VMPR and shorter HRT, there is the absence of substrate overloading, which is known to result in declined performance and reactor failure [53].

#### 2.1.3. Impact of a Fill-and-Draw Inoculum

Mono-digestion at 35 °C required an active inoculum drawn as digestate at day 120 from another GM-based anaerobic digester previously operating at 45 °C. A combination of digestion conditions, such as digestate recirculation as downstream inoculum, allowed for the prior acclimation of microbes to the specific substrate type, resulting in reduced lag time (Figure 1) [54]. Shi et al. [55] observed that, in the treatment of lignocellulosic biomass, a prior acclimation period of the microbes contained in the inoculum was necessary for immediate biogas production in digesters. Additionally, a sufficient contact time reduced the magnitude of the microbial response to potentially toxic compounds, namely excessive levels of ammonia, volatile fatty acids, and heavy metals that can exert bacteriostatic and even bactericidal effects [56,57,58].

### 2.2. Digestate Characterisation after Treatment

#### 2.2.1. Chemical Oxygen Demand (COD) and Total Kjeldahl Nitrogen (TKN)

The treatment recorded an overall removal of 97 g CODt kg^−1^, with a daily removal rate of 2.31 g CODt kg^−1^ d^−1^. A total of 82.75% of the degraded organic matter was converted to methane.

The TKN and COD values in the effluent were used to approximate the actual nutritional quality of the digestate during reactor runs because not all the nitrogen and carbon present in the feedstock were available for digestion. The COD/N ratio was 28.6/1; following the treatment cycle, nitrogen removal reached 66.67% (Table 1).

Gil et al. [59] found that methane production directly correlated with nitrogen removal and that nitrogen accumulation was inhibitory to methanogenesis. Excess nitrogen can lead to excessive ammonia formation, resulting in toxic effects and ultimately reactor failure [60]. A balanced COD/N ratio is required to avoid extremes of nutrient limitation or ammonia toxicity resulting from low and high levels of nitrogen, respectively. In summary, the low CODt and high nitrogen removal provided a suitable nutritional balance to microbes; thus, it was conducive to an increased methane yield [50].

#### 2.2.2. pH

The higher final pH (8.21 ± 0.18) recorded could be attributed to the hydrolytic action on polymers proportional to the hydraulic retention time, resulting in lower cumulative CODt removal (Table 1). However, the accumulation of volatile fatty acids ultimately produced mild acidification in relation to the physicochemical balance at the beginning of the digestion [61]. Additionally, organically-bound nitrogen present in the feedstock can be converted to ammonia that potentially behaved as a base, buffering the volatile acids produced by hydrolytic and acidogenic microbes. These redox reactions adequately neutralised pH fluctuations to maintain favourable metabolic conditions [62].

#### 2.2.3. Electrical Conductivity (EC) and Salinity

EC in the effluent was relatively stable, having lowered to 28.2 ± 1.70 mS cm^−1^ (9.03% reduction in the initial value). Previous studies correlated high conductivity to methane production and even used conductivity as a predictor for reactor performance [63,64].

Salinity increased to 9.0% in the effluent (Table 1). Microbe-driven solubilisation of polymers is optimal in the salinity range 0.23–0.35 g/L [65]. Throughout treatment, the ionic species and soluble minerals that were initially in the granular state and were organically bound were released into the medium, further raising the salinity. As evidenced by the effluent profiles, salinity positively correlated with a higher SMY.

### 2.3. Kinetic Results

A kinetic study was carried out by fitting both the first-order and the modified Gompertz models to the experimental data. The predictive parameters and corresponding values of trial results are shown in Table 2. The differences between the measured and the predicted methane production were 5.17% and 5.39% for the first-order kinetic and the modified Gompertz, respectively. In addition, the test statistic root-mean-square deviation (RMSD) was 0.009 m^3^ CH_4_ kg^−1^ VS and 0.003 m^3^ CH_4_ kg^−1^ VS for the first-order kinetic and the modified Gompertz, respectively. In general, the lower the RMSD value, the better the goodness-of-fit.

Both models closely fitted the experimental data. Statistically, the modified Gompertz model would be the better agreement for data fit considering the lower RMSD over the treatment period of 42 days (Table 2). Nevertheless, based on trends in differences between the experimental and predicted methane production, the modified Gompertz model appeared appropriate for short-term treatment where the lag time exerts a greater effect on the maximum cumulative methane produced due to the inhibitory effects of the long-chain fatty acids [29]. The first-order kinetic model improves the fit of data for the long-term because the effect of the initial lag becomes progressively muted as the cumulative methane production rises, hence the apparent linearisation of the methane curve in the final stage of biogas production (Figure 1).

Donoso-Bravo et al. [66] stated that the abundant availability of readily digestible compounds drives predictive simulations towards first-order kinetic mathematical models. However, as observed previously, when cumulative methane production slows down, GM-based progress curves steadily rebalance to the modified Gompertz model [29]. The high content of potassium and lipids in wastes may result in prolonged lag time [54].

Waste management strategies aimed at mitigating the extent of the lag phase to reach stable performance during AD often involve a lengthy preparatory acclimation stage of wastes; a fill-and-draw treatment plant configuration (waste recirculation as subsequent inoculum) to feed the digesters downstream [54], slurry mixing during operation [50], and the lowering of the substrate-to-inoculum ratio [47,67].

### 2.4. Bacterial Community Structure

**Figure 3:**
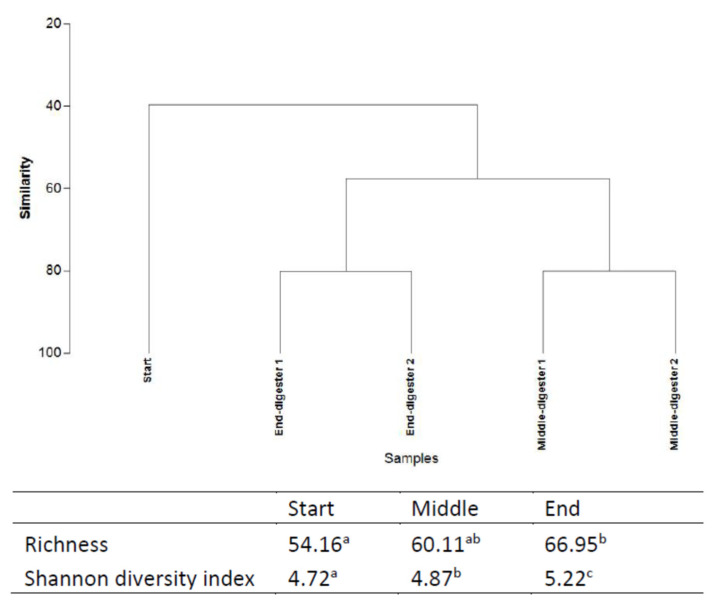
Cluster analysis (UPGMA method), Richness and Shannon diversity indices based on 16S rDNA sequencing at different time points (start, middle, and end) during the anaerobic digestion (means in the same column with the same letter are not significantly different at *p* < 0.05). Treatments were conducted in a duplicate anaerobic system (digesters 1 and 2) over 42 days at 35 °C. Letters “a” “b” “c” are indicative of the statistical significance level (where variables are of the same letter, the difference between the means is not statistically significant).

Relating AD performance and microbial community function, molecular analysis was performed through amplicon-based sequencing based on 16S rDNA from digestate samples taken at start-up, during, and at the termination of the digestion [68]. The impact of the selection pressures imposed by the operating parameters and possible functional synergies between the organisms of the bacteriome were evaluated. Both richness and diversity of the bacterial community both during and at the end of the digestion period were significantly increased from day 0 (Figure 3). The Richness index progressively increased from 54.16 (initially) to 60.11 (middle), culminating at 66.95 at the conclusion of treatment. In terms of species richness (total number) and evenness (relative abundance), both correlate to bacterial metabolism rates in ecosystems [68]. For example, in a microcosm study of denitrifying bacterial communities, Wittebolle et al. [69] concluded that both high richness and evenness translated to high metabolic rate (denitrification) among bacterial communities in the presence of salt stress. However, an inverse metabolic trend was observed in the present mono-digestion of GM. Higher richness (66.95) and diversity (5.22) at the termination, in comparison to the middle (richness = 60.11; diversity = 4.87; VMPR = 2.0503 L CH_4_ L_Work_^−1^ d^−1^) of the digestion correlated to a lower final VMPR (0.7065 L CH_4_ L_Work_^−1^ d^−1^; Figure 2). Inhibition of methane production is common in AD operations due to the build-up of volatile fatty acids resulting from the faster kinetics in the hydrolysis, acidogenesis, and acetogenesis stages positioned upstream of the generally slower methanogenesis stage, resulting in a drop of final VMPR values, despite increased microbial richness and evenness at the end of the digestion [12].

### 2.5. Bacterial Community Dynamics

**Figure 4:**
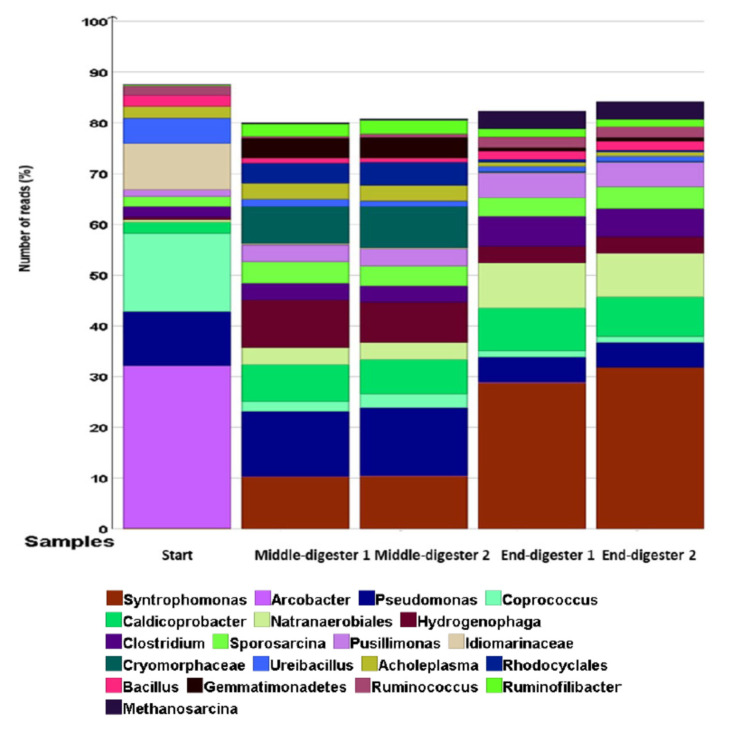
Relative abundance of bacteria at the genus level based on 16S sequencing at different time points (start, middle, and end) of digestion. Treatments were conducted in a duplicate anaerobic system (digesters 1 and 2) over 42 days at 35 °C. The important genera are shown in the legend.

The initial bacterial community was predominantly composed of aerobes that were mainly from *Arcobacter* (32% of all reads), *Pseudomonas* (10% of all reads), *Idiomarinaceae* (10% of all reads), *Ureibacillus* (5% of all reads), and an amalgamation of other aerobes (*Hydrogenophaga*, *Pusillimonas*, *Cryomorphaceae*, *Gemmatimonadetes*), making up <2% of the total reads. On day 0, the key anaerobes were from the genera of *Coprococcus* (16% of all reads reads), *Caldicoprobacter* (2% of all reads), *Clostridium* (2% of all reads), *Sporosarcina* (2% of all reads), *Bacillus* (2% of all reads), *Ruminococcus* (2% of all reads), and other *Natranaerobiales*; *Rhodocyclales* accounted for <1% of all reads (Figure 4). However, the bacterial profile moved towards anaerobes by the end of the digestion (Figure 4).

Because of the depletion of oxygen in the headspace during treatment, there was a reduction in aerobic bacterial populations, especially *Pseudomonas*, amongst others [70]. Consequently, no detectable aerobic emergent enteropathogen genus of *Arcobacter* were observed [71]. Similarly, the halophile genus of the Gram-negative *Idiomarinaceae* decreased to <0.5% of all reads; the genus *Ureibacillus* reduced to 1% of all reads [72,73]. However, digestion conditions became increasingly favourable to the obligate anaerobes of the genus *Caldicoprobacter*, increasing to 9% of all reads during the treatment. Other obligate anaerobes of the genus *Natranaerobiales* increased from <0.5 to 3% of all reads during digestion. Most notably, the population of anoxygenic photoheterotrophic *Rhodocyclales* increased from <0.1% to 5% of all reads (Figure 4) [74].

At the end of the digestion, the molecular profile was attuned to microbial physiologies better suited to anaerobic conditions. The anaerobic non-phototrophic *Syntrophomonas* were undetectable in the beginning (<0.1% of all reads) and increasing during the digestion (10% of all reads), reaching 29% of all reads at the end of treatment. In addition, the relative abundance of the Gram-positive population of anaerobic *Sporosarcina* doubled (from 2% initially, to 4% of all reads). In the same way, an increase in overall richness was observed among anaerobic *Clostridium* (from 2% of all reads initially, to 6% of all reads), *Natranaerobiales* (from 2% of all reads, initially, to 9% of all reads), and *Caldicoprobacter* (from 2% of all reads, initially, to 10% of all reads). Methanogenic *Archaea* from the genus *Methanosarcina* represented a clearly identifiable bacterial group at the termination of digestion (from <0.1% of all reads, initially, to 5% of all reads). There was a reduction of the pathogenic *Arcobacter* to trace levels by the end of the treatment.

In terms of possible symbiotic relationships, the genus *Coprococcus* is classified as a group for butyrate-producing bacteria [75]. Butyrate, a fermentation intermediate, can be utilised by anaerobic butyrate-degrading bacteria of the *Syntrophomonas* genus [76,77]. In addition to butyrate, other volatile fatty acids can be digested by *Syntrophomonas* to produce hydrogen and acetate in a syntrophic dependence on hydrogen-utilising bacteria to reduce carbon dioxide to methane [77]. Concurrently, acetate can be utilised by acetoclastic methanogens such as *Methanosarcina*, the terminal metabolic group. In addition, the anaerobic genus of the Gram-positive *Ruminococcus* is known for cellulolytic, pectinolytic and hemicellulolytic activity. *Ruminococcus* species are capable of degrading organic polymers as their sole carbon source, causing the release of glucose monomers or metabolites for further digestion by adjacent microorganisms [78]. Considering the important hydrolytic functions of *Ruminococcus*, which release energy from complex polysaccharides to microbes in the microbiome, these anaerobes are regarded as key players in anaerobic ecosystems. Both hydrolytic and fermentative pathways have been reported for organisms assigned to the *Firmicutes* and *Bacteroidetes* phyla [68]. Cluster analyses revealed richness of 60% and 19% reads for *Firmicutes* and *Bacteroidetes*, respectively. Additionally, *Clostridium* species were documented in terms of lignocellulosic hydrolysis [35]. Whilst these important microbial groups mediate the upstream metabolic stages of AD for syntrophic electron flow on to *Archaea*, there may be an accumulation of fermentation products which may in turn inhibit methane production, resulting in a decreased VMPR trend (Figure 2) [12].

It can also be noted from Figure 4 that aerobic *Hydrogenophaga* and *Pusillimonas* genera from the *Proteobacteria* phylum displayed trends of increasing microbial richness from the initial values, despite depleting oxygen levels because of chemoorganotrophic or chemolithoautrophic competence, allowing for oxidation of hydrogen as energy source and CO_2_ as carbon source [79,80,81]. During the mono-digestion of GM, the CO_2_ concentration reached a peak of 23.6% of total biogas volume on day 5 of digestion, lowering to 11.4% and 11.0%, on day 20 and 42, respectively. Moreover, the physicochemical composition of the mixed GM and inoculum resulted in highly saline and alkaline conditions suitable for the growth of haloalkaliphiles such as anaerobic *Natranaerobiales* [82]. Conversely, other known aerobic halophiles such as *Idiomarinaceae* and *Cryomorphaceae* could not take advantage of substrate utilisation, likely due to the prolonged unfavourable anoxic environment for growth, and thus they were progressively sieved out of digestion (Figure 4) [73,83].

### 2.6. Valorisation from Winery Residues

Depending on the magnitude of the grape crush, an economic model, Table 3, was projected from the following parameters: (i) electrical and calorific efficacies of 35% and 40%, respectively [29]; (ii) 362.3 g of CO_2_ for each kWh produced, in metric tonnes [29]; (iii) Australian average electricity and gas prices of EUR 0.17/kWh and EUR 0.13/MJ, respectively, excluding off-peak tariffs and user discounts [84]; (iv) EUR 9.52 per ACCU, unit by the Clean Energy Regulator as of September 2019; each ACCU issued represents one metric tonne of carbon dioxide equivalent (Mt CO_2_-e) stored or avoided by a project [85].

In order to achieve the required 35 °C temperature regime for digestion, some form of energy input, such as biogas combustion from a preceding reactor run, would be envisaged [86]. Wineries and GM-based distilleries routinely have an abundant seasonal biomass that can be channelled to anaerobic treatment. An on-site development would reduce collection, transport, and delivery costs to the energy-conversion site. In addition, a fill-and-draw AD operational model for a winery-wide scale, involving the reuse of digestate to drive downstream digesters configured as industrial tubular reactors, would further reinforce the tenets for an economy of proximity. Improvements in methane production efficiencies from winery residues would further offset the investment and running costs of AD [13,87].

Taking into consideration the escalating year-on-year electricity and gas tariffs as well as technological advancements in energy conversion systems, wineries would increasingly capture economic value when investing in the anaerobic treatment of GM as an integrated step in a waste valorisation strategy. Additionally, the CO_2_ avoidance achieved during GM treatment can be further capitalised on carbon credit trading platforms (Table 3). These proactive measures would translate into energy cost-savings and self-reliance, as well as the exporting of energy surplus to the power grid, seasonally.

## 3. Materials and Methods

### 3.1. Substrate Characterisation and Analytical Methods

Dried GM that had undergone prior distillation for alcohol recovery was sourced from Tarac Technologies, Australia. Grape marc was milled with the use of a household blender to obtain mass homogeneity with particle sizes of 1–2 mm [47,88] and stored at 4 °C until use [67]. The methanogenic inoculum was sampled, in a fill-and-draw approach, from an active 120-day laboratory-scale digester of composition 3/1 grape marc and cheese whey, respectively, operating at 45 °C. The characterisation parameters reported in Table 4 were determined, in triplicate, on the digestion content before and after incubation. The solids, COD, and the total Kjeldahl nitrogen (TKN) were determined according to standard methods [89]. Briefly, the total chemical oxygen demand (CODt) was determined by sample digestion with manufacturer-provided reagents in a HACH DRB 200 heating block with values read on a HACH DR 900 colorimeter. The soluble COD (CODs) in the liquid fraction was determined by first spinning down samples in a centrifuge at 13,000 rpm for 5 min and then determining the COD of the supernatant, as described previously. Total solids (TS) were determined by subjecting 100 g of samples to 105 °C dry heating in an oven for 24 h, cooled in a desiccator and weighed followed by incubation in a furnace at 550 °C for 2 h for determination of volatile solids (VS) with an intervening cooling down before weighing. For bacterial analysis, 5 g of digestate was also sampled during the digestion as well as at the beginning and end. HANNA Instruments edge^pH^ was used to measure pH. Salinity and conductivity were determined by means of a Compact Salt Meter (LAQUAtwin-Salt-11, HORIBA Scientific, Kyoto, Japan) and a Compact Conductivity Meter (LAQUAtwin-CC-11, HORIBA Scientific), respectively.

### 3.2. Substrate-to-Inoculum Ratio (SIR)

Ma et al. [48] reported that a high SIR resulted in a considerable lag, accumulation of volatile fatty acids, and low pH. In contrast, reactors operating at lower SIR values were defined by increased microbial activity, high volumetric methane productivity, high daily methane yield, and retracted lag [48]. Motte et al. [47] concluded that low SIR exerted a substantial positive impact on the start-up phase, resulting in the early production of methane in the anaerobic treatment of lignocellulosic substrates. Previously, in the co-digestion of solid winery wastes and agri-industrial dairy wastes, Kassongo et al. [90] used 10:1 SIR. Understanding the importance of SIR on reactor performance, the study of the dynamic effect of methane production further lowered the SIR to 10:3 for the mesophilic mono-digestion of marc.

### 3.3. Methane Production and Performance Monitoring

The treatment conditions required an inoculum previously acclimatised to GM; the SIR was at 10:3 (wet weight basis) and the working volume (V_W_) reached 1.3 kg. Before digestion at 35 °C, GM and inoculum were thoroughly mixed; there was no subsequent mixing or substrate feeding during digestion.

The batch mode treatment configuration closely mimics the characteristics of an ideal plug-flow reactor comparable to industrial tubular reactors for the treatment of solid-state organic wastes, with an assumed reaction rate proportional to the reactant concentration [87]. The study was conducted in a W8 Anaerobic Digester (Armfield, Ringwood, UK); the temperature was raised to 35 °C in a single step [50].

Biogas was determined by water displacement in cylinders paired with the reaction vessels [50]. The biogas composition was measured in a GEM2000 Landfill Gas Analyser (Geotech, Coventry, UK). The specific methane yield (SMY) of the digestion setups corresponded to the cumulative methane fraction of the biogas as a function of the volatile solids (VS) as digestion progressed; SMY is expressed as m^3^ CH_4_ kg^−1^ VS. Blank assays, without substrate, were conducted for the determination of the residual methane potential of the inoculum [19].

### 3.4. Volumetric Methane Productivity Rate (VMPR)

The VMPR is the daily methane produced (L) per unit working volume of the reactor. VMPR is expressed as L CH_4_ L_Work_^−1^ d^−1^ [48,91]. The average VMPR that describes the whole digestion process is calculated according to Equation (1):

(1) $$VMPR=V1/\left(V2\;\cdot T_{80}\right)$$

where *V*2 is the reactor working volume (L) and *T*_80_ is the shortest technical digestion time (d) calculated according to the time for the cumulative methane volume to achieve 80% of *V*1.

### 3.5. Biodegradability Index (BI)

The *BI*, in %, was calculated using the theoretical methane potential (*B*_0_) determined based on the COD removed and that a maximum 350 mL of methane emissions can be extracted from 1 g of COD daily, using Equation (2):

(2) $$BI=\left(\frac{SMY}{B_{0}}\right)100$$

### 3.6. Regression Models for Data Fit

To describe the biomethanation process, non-linear regressions were utilised [29,92]. The degradation of organics was assumed to be patterned along with a first-order rate of decay due to the microbial role in the fermentation process; thus, the first-order equation (Equation (3)) is as follows:

(3) $$B\left(t\right)=B_{0}\;\left[1-\exp\left(-kt\right)\right]$$

where *B*(*t*) is the cumulative methane volume (m^3^ CH_4_ kg^−1^ VS) at a digestion time *t* (d), *B*_0_ is the methane potential of the substrate material (m^3^ CH_4_ kg^−1^ VS), *k* is the first-order disintegration rate constant (d^−1^), and *t* is the digestion time (d).

To estimate the lag phase, a modified Gompertz model was simulated (Equation (4)):

(4) $$B\left(t\right)=B_{0}\;.\exp\left\{-\exp\left[\left(\frac{R_{m}.\;exp}{B_{0}}\right)\left(\lambda -t\right)+1\right]\right\}$$

where *R_m_* is the maximal methane production rate (m^3^ CH_4_ kg^−1^ VS d^−1^) and *λ* is the lag phase (d); all mathematical models were simulated with the Solver tool of Microsoft Office Excel.

### 3.7. Microbial DNA Isolation and Sequencing

An aliquot (0.25g) of digestate samples (in duplicate) were processed for DNA extraction using a PowerSoil^®^ DNA Isolation Kit (Mo Bio Laboratories, Carlsbad, CA, USA). Purified DNA samples were sent to AGRF (Westmead, Australia) for Next Generation Sequencing in Illumina’s MiSeq platform. Primer, PCR, and library preparations were prepared according to AGRF guidelines. Illuminabcl2fastq 2.20.0.422 pipeline was then used to generate the sequence data. Diversity profiling analysis was performed with QIIME 2 2019.7 [93]. The demultiplexed raw reads were primer trimmed and quality filtered using the cutadapt plugin followed by denoising with DADA2 (via q2-dada2) [94]. Taxonomy was assigned to ASVs using the q2-feature classifier classify-sklearn naïve Bayes taxonomy Taxonomic classifier [95]. Results were then analysed on PRIMERS7 and MEGAN 6 in terms of cluster analysis, Shannon diversity, and richness. Community composition was conducted at the genus level using Bray–Curtis similarity via non-metric multidimensional scaling.

### 3.8. Statistical Analyses

Mean values ± standard error were reported for the study. Experimental data were processed in the statistical computer programs GraphPad Prism v. 7.02 and SPSS v. 23. Statistical significance at the level of *p* < 0.05 was evaluated by multivariate one-way ANOVA. Means were separated using Tukey’s HSD posthoc parametric test, where the F-value was significant, by comparing the differences between means of values [96].

## 4. Conclusions

Based on the experiments conducted with grape marc, there was a cost-effective regulation of operational parameters such as inoculum sourcing, substrate-to-inoculum ratio, digester working volume, and hydraulic residence time, collectively impacting on the overall bioenergy production profile and the attainable remediation levels of winery residues. A fill-and-draw approach for digestate recirculation as an incoming downstream inoculum allowed for sufficient acclimation time between microbes and the substrate type, resulting in a shortened lag in subsequent treatment setups, additionally reducing operating costs and increasing treatment efficiency. A higher inoculum dose (10:3 SIR) in the reaction mixture naturally increased the bio-catalytic density. These operational adjustments, without requirements for exogenous synthetic enhancers, improved the inherent catalytic capabilities of the digestion cycle and provided abundant readily digestible compounds for the short-term, and slowly biodegradable substrates for the long-term treatments. The results were in line with previous studies where operational regulation positively impacted anaerobic treatment.

There was sanitisation of the digestate by the removal of known potential zoonotic *Arcobacter* by the end of digestion. Additionally, consortia of anaerobic hydrolytic and fermentative *Firmicutes* and *Bacteroidetes* were core microorganisms in the digester, representing nearly 80% of the microbial community. *Archaea* from the *Methanosarcina* genus were credited with methane production through acetoclastic methanogenesis. The maximum cumulative methane productivity achieved after the grape marc treatment was 0.145 m^3^ CH_4_ kg^−1^ VS. Economic simulations for valorisation from grape marc through investments in anaerobic digestion technology showed potential for a seasonal revenue stream for wineries through energy savings and commercialisation of excess energetic equivalents and carbon credits, further entrenching an economy of proximity.

Future research will explore the impact of lower substrate-to-inoculum ratios on reactor performance and target progressive microbial community engineering during anaerobic digestion of grape marc.

## Figures and Tables

**Table 1:** Characteristics of the grape marc effluent after mono-digestion in unmixed treatment conditions over 42 days at 35 °C. Values are presented as mean ± standard error.

	Value
**Operational Conditions**	
SIR	10:3
V_W_ (kg)	1.30
HRT (d)	42
**Effluent Characteristics**	
Total solids (%)	33.75 ± 1.223
Volatile solids (%)	27.39 ± 1.167
Total chemical oxygen	
demand (CODt) (g kg^−1^)	126 ± 4.95
Conductivity (mS cm^−1^)	28.2 ± 1.70
Salinity (%)	9.0 ± 0.0
Total Kjeldahl-N (g kg^−1^)	4.40 ± 0.31
COD/N	28.6/1
pH	8.21 ± 0.18
**Yields**	
CODt removed (g kg^−1^)	97.0
CODt removal efficiency (%)	43.50
SMY (m^3^ CH_4_ kg^−1^ VS_fed_)	0.145 ± 0.00
(m^3^ CH_4_ kg^−1^ COD_removed_)	0.289
BI (%)	82.75

V_W_, working volume; HRT, hydraulic retention time; BI, biodegradability index.

**Table 2:** Kinetic parameters for the grape marc treatment based on the predictive non-linear first-order kinetic and the modified Gompertz models of the process parameters at 35 °C over an incubation period of 42 days.

Simulation	Unit	Value
***First-order kinetic model***		
B_0_	m^3^ CH_4_ kg^−1^ VS	4.468
k	d^−1^	0.001
Sum of squared deviations (SSD)	—	0.004
Root-mean-square deviation (RMSD)	m^3^ CH_4_ kg^−1^ VS	0.009
Measured methane yield	m^3^ CH_4_ kg^−1^ VS	0.144
day 42		
Predicted methane yield	m^3^ CH_4_ kg^−1^ VS	0.152
day 42		
Difference between measured and predicted	%	5.177
methane yield (in absolute value)		
***Modified Gompertz model***		
B_0_	m^3^ CH_4_ kg^−1^ VS	0.143
λ	d	6.953
R_m_	m^3^ CH_4_ kg^−1^ VS d^−1^	0.006
Sum of squared deviations (SSD)	—	0.001
Root-mean-square deviation (RMSD)	m^3^ CH_4_ kg^−1^ VS	0.003
Measured methane yield	m^3^ CH_4_ kg^−1^ VS	0.144
day 42		
Predicted methane yield	m^3^ CH_4_ kg^−1^ VS	0.136
day 42		
Difference between measured and predicted	%	5.392
methane yield (in absolute value)		

**Table 3:** An economic simulation of the valorisation from available biomass based on the size of a winery in tandem with a distillery operation (totals may not sum due to rounding). The currency used is EUR (Euro).

		Winery Size		
		Small	Medium	Large
Grape crush				
(10^3^ t season^−1^)		0.2	1	5
Grape marc availability				
(10^3^ t season^−1^)		0.04	0.2	1
Energetic equivalents	Primary	8.63	43.1	216
(MWh season^−1^)	Electrical	3.02	15.1	75.5
	Thermal	3.43	17.3	86.3
CO_2_ emissions avoided				
(Mt CO_2_-e season^−1^)		2.34	11.7	58.6
	Electrical	0.51	2.57	12.9
Economic returns	Thermal	0.45	2.25	11.2
(EUR 1000 season^−1^)	Carbon credits	0.02	0.11	0.56
	TOTAL	0.98	4.93	24.7

**Table 4:** Analytical characterisation of the grape marc-based reactor setup and inocula at reactor start-up before treatment at 35 °C; data reported as mean ± standard error.

	Unmixed Feedstock		Reactor
Parameter	(Grape Marc)	(Inoculum)	(Combined)
Total solids, TS (%)	38.7 ± 1.51	21.5 ± 0.07	31.9 ± 2.02
Volatile solids, VS (%)	24.1 ± 0.54	15.1 ± 1.82	19.4 ± 1.23
Total COD, CODt (g kg^−1^)	223 ± 16.3	101 ± 7.23	223 ± 11.5
Soluble COD, CODs (g kg^−1^)	47.5 ± 12.0	13 ± 0.0	20 ± 3.0
Electrical conductivity, EC (mS cm^−1^)	15.0 ± 0.20	15.6 ± 0.12	30.9 ± 0.49
Salinity (%)	5.20 ± 0.32	9.75 ± 0.10	7.0 ± 1.4
pH	9.19 ± 0.00	7.91 ± 0.16	9.03 ± 0.11
Total Kjeldahl-N (g kg^−1^)	51.8 ± 0.76	2.42 ± 0.32	12.6 ± 0.10

## Data Availability

The data presented in this study are available on request from the corresponding author.

## Sample Availability

Samples of the compounds in this article are not available from the authors.
